# Cell Type-Specific Human APP Transgene Expression by Hippocampal Interneurons in the Tg2576 Mouse Model of Alzheimer’s Disease

**DOI:** 10.3389/fnins.2019.00137

**Published:** 2019-02-22

**Authors:** Corinna Höfling, Emira Shehabi, Peer-Hendrik Kuhn, Stefan F. Lichtenthaler, Maike Hartlage-Rübsamen, Steffen Roßner

**Affiliations:** ^1^Paul-Flechsig-Institute for Brain Research, Leipzig University, Leipzig, Germany; ^2^Institute of Pathology, Technical University of Munich, Munich, Germany; ^3^Deutsches Zentrum für Neurodegenerative Erkrankungen, Munich, Germany; ^4^Munich Cluster for Systems Neurology (SyNergy), Munich, Germany; ^5^Neuroproteomics, School of Medicine, Klinikum Rechts der Isar, Technical University of Munich, Munich, Germany; ^6^Institute for Advanced Study, Technical University of Munich, Garching, Germany

**Keywords:** Alzheimer’s disease, animal model, amyloid precursor protein, hippocampus, interneuron, calcium-binding proteins

## Abstract

Amyloid precursor protein (APP) transgenic animal models of Alzheimer’s disease have become versatile tools for basic and translational research. However, there is great heterogeneity of histological, biochemical, and functional data between transgenic mouse lines, which might be due to different transgene expression patterns. Here, the expression of human APP (hAPP) by GABAergic hippocampal interneurons immunoreactive for the calcium binding proteins parvalbumin, calbindin, calretinin, and for the peptide hormone somatostatin was analyzed in Tg2576 mice by double immunofluorescent microscopy. Overall, there was no GABAergic interneuron subpopulation that did not express the transgene. On the other hand, in no case all neurons of such a subpopulation expressed hAPP. In dentate gyrus molecular layer and in stratum lacunosum moleculare less than 10% of hAPP-positive interneurons co-express any of these interneuron markers, whereas in *stratum oriens* hAPP-expressing neurons frequently co-express these interneuron markers to different proportions. We conclude that these neurons differentially contribute to deficits in young Tg2576 mice before the onset of Abeta plaque pathology. The detailed analysis of distinct brain region and neuron type-specific APP transgene expression patterns is indispensable to understand particular pathological features and mouse line-specific differences in neuronal and systemic functions.

## Introduction

Alzheimer’s disease (AD) is histopathologically characterized by the formation of intracellular neurofibrillary tangles, principally made of tau protein and extracellular amyloid deposits composed of Abeta peptides that deposit in the parenchyma and in the vessel walls. These Abeta peptides are cleavage products generated by proteolytical processing of the amyloid precursor protein (APP) by the β-secretase BACE1 (Lichtenthaler et al., 2011; Vassar et al., 2014) and the γ-secretase complex composed of presenilin-1 or -2, nicastrin, APH-1, and PEN-2 (Kimberly et al., 2003). Abeta plaques represent a final aggregation state of Abeta peptides and are used for *post mortem* AD diagnosis but can be also detected by positron emission tomography imaging in patients (Barthel and Sabri, 2017; Salloway et al., 2017).

A substantial gain of knowledge on mechanisms of amyloid pathology in AD was achieved by the analyses of transgenic mouse models overexpressing human APP (hAPP) with disease-promoting mutations that lead to early-onset AD in humans (Ameen-Ali et al., 2017; Sasaguri et al., 2017). However, these animal models differ significantly regarding the onset of pathology, spatial appearance of Abeta deposits, neuronal loss and deficits in learning and memory tasks as reviewed by Höfling et al. (2016) which hampers drawing general conclusions on defined pathogenic functions of Abeta peptides. Thus, a detailed analysis of the brain region and cell type-specific transgene expression patterns is indispensable to understand pathogenic mechanisms in each animal model. A very well characterized and frequently used transgenic model is the Tg2576 mouse established by Hsiao et al. (1996). These mice overexpress hAPP harboring the Swedish double mutation KM670/671NL and develop Abeta deposits starting in entorhinal cortex followed by hippocampus at around 11 months of age (Hsiao et al., 1996; Kawarabayashi et al., 2001; Hartlage-Rübsamen et al., 2011).

Interestingly, these mice display impaired hippocampus-dependent spatial learning, working memory, and contextual fear conditioning already at 6 months of age (King and Arendash, 2002), which is well before extracellular plaques appear in the brains of these mice. The deficiencies in spatial learning and memory consolidation are of particular interest since they resemble clinical aspects of AD patients such as disturbed spatial orientation (Kumar et al., 2015) and altered neuronal network activity (Allen et al., 2007; Brier et al., 2012; Sheline and Raichle, 2013). In that regard dendritic spine loss in the Tg2576 CA1 region (Lanz et al., 2003) and a decline in long-term potentiation (LTP) in dentate gyrus (DG) after performant path stimulation (Jacobsen et al., 2006) already detectable at 5 months of age point towards a pathogenic role of soluble, oligomeric Abeta prior to Abeta plaque formation. Additionally, using resting-state functional Magnetic Resonance Imaging (MRI), a hypersynchrony of functional connectivity in the hippocampus of 5-month-old Tg2576 mice was demonstrated (Shah et al., 2016) suggesting increased excitatory and/or reduced inhibitory neuronal activity. These pathogenic aspects have been frequently related to Abeta oligomer formation. However, a series of recent studies from different laboratories demonstrates that such disturbances may occur independently of Abeta formation. In particular, there appears to be a causal link between early pathogenic events including lysosomal autophagic pathology, hyperactivity in lateral entorhinal cortex, early brain network alterations in the CA1/subiculum and the generation of intracellular APP C-terminal fragments not cleaved by γ-secretase (Lauritzen et al., 2012, 2016; Xu et al., 2015; Mondragón-Rodríguez et al., 2018). In addition, the AD-related endosome dysfunction in Down syndrome was demonstrated to be independent of Abeta generation but to rely on the BACE1-catalyzed formation of the APP C-terminal fragment C99 (Jiang et al., 2010).

However, both the generation of soluble human Abeta and of C-terminal hAPP fragments require transgenic hAPP expression. In that regard, it is a still unaddressed question which specific neuronal populations are affected by hAPP overexpression in Tg2576 hippocampus. Theoretically, either neurons expressing the hAPP transgene or those exposed to extracellular Abeta assemblies might be specifically affected. Employing a novel, hAPP-specific rat monoclonal antibody we have recently demonstrated transgene expression by virtually all CA1 to CA3 pyramidal neurons and by scattered GABAergic interneurons throughout all hippocampal layers, but not by DG granule cells (Höfling et al., 2016). These interneurons represent a small proportion of hippocampal neurons but generate and control the rhythmic output of large populations of principal cells and modulate LTP (Chen et al., 2010; Huh et al., 2016), neurogenesis (Krezymon et al., 2013; Verdaguer et al., 2015), Abeta pathology (Ramos et al., 2006) as well as learning and memory (Perez-Cruz et al., 2011; Schmid et al., 2016). Thus, the observed early alterations in hippocampal network activity may be due to functional impairment of inhibitory GABAergic hippocampal interneurons expressing the hAPP transgene in Tg2576 mice. These GABAergic hippocampal interneurons can be subdivided based on shape, localization in defined hippocampal layers and the co-expression of specific calcium-binding proteins (CBPs) such as parvalbumin, calbindin, and calretinin as well as the peptide hormone somatostatin (Mátyás et al., 2004).

In order to identify hippocampal interneurons expressing the hAPP transgene in Tg2576 mice, double immunofluorescent labelings detecting hAPP and the CBPs and somatostatin were performed followed by quantitative stereological analyses. The data reported identify defined hippocampal populations of interneurons as cellular source for Abeta generation which may affect the neurons expressing the transgene themselves or anatomical target areas where Abeta peptides are released from these neurons.

## Materials and Methods

### Animals

In order to identify GABAergic interneurons expressing the hAPP transgene we used 3-month-old female wild type (*N* = 3) and female Tg2576 mice *(N* = 3) on C57BL/6xSJL background in which transgene expression is driven by the hamster prion protein promoter (Hsiao et al., 1996). Female mice were chosen because they develop Abeta pathology earlier than males and display a higher amyloid load than males at the same age (Callahan et al., 2001) and are, therefore, used more often in experimental studies. Animals were housed at 23°C at 12 h day/12 h night cycles with food and water *ad libitum* in cages that contained red plastic houses (Tecniplast) and shredded paper flakes to allow nest building. All experimental protocols were approved by Landesdirektion Sachsen, license T28/16 and all methods were carried out in accordance with the relevant guidelines and regulations.

### Tissue Preparation

Mice were sacrificed by CO_2_ inhalation and perfused transcardially with 50 ml 0.9% saline followed by perfusion with 80 ml 4% paraformaldehyde in PBS (0.1 M; pH 7.4). The brains were removed from the skull and postfixed by immersion in the same fixative overnight at 4°C. After cryoprotection in 30% sucrose in 0.1 M PBS for 3 days, coronal sections (30 μm) were cut at the level of Bregma -2.70 mm to Bregma -3.16 mm on a sliding microtome and collected in 0.1 M PBS.

### Immunohistochemistry

In order to reveal the neuron type-specific labeling generated by the rat monoclonal anti-hAPP antibody 1D1 (15 μg/ml) in hippocampus of Tg2576 mice triple immunofluorescent labeling in combination with antibodies directed against the CBPs parvalbumin (rabbit anti-parvalbumin; Swant; 1:4,000), calbindin (rabbit anti-calbindin; Swant; 1:1,000), calretinin (rabbit anti-calretinin; Swant; 1:1,000) and against somatostatin (goat anti-somatostatin; Santa Cruz; 1:200) was performed. To allow normalization for total neuronal numbers a mixture of mouse anti-NeuN/anti-HuCD (1:1000 each; Invitrogen/Millipore) was added to the incubation cocktails. Brain sections were incubated with cocktails of primary antibodies overnight at 4°C. On the next day, sections were washed three times with TBS and were then incubated with cocktails of Cy3-conjugated donkey anti-rat (1:400; Dianova) and Cy2-conjugated donkey anti-rabbit or donkey anti-goat (1:400; Dianova) and Cy5-conjugated donkey anti-mouse (1:400; Dianova) for 60 min at room temperature. This procedure resulted in red labeling of hAPP-positive neurons, in green labeling of the respective interneuron types and in blue labeling of all neurons. After washing, sections were mounted onto glass slides and coverslipped.

### Microscopy

Confocal laser scanning microscopy (LSM 510, Zeiss, Oberkochen, Germany) was performed to reveal co-localization of hAPP with parvalbumin, calbindin, calretinin, and somatostatin in hippocampus. For Cy2-labeled interneuron markers (green fluorescence), an argon laser with 488 nm excitation was used and emission from Cy2 was recorded at 510 nm applying a low-range band pass (505–550 nm). For Cy3-labeled hAPP (red fluorescence), a helium-neon-laser with 543 nm excitation was applied and emission from Cy3 at 570 nm was detected applying high-range band pass (560–615 nm). Photoshop CS2 (Adobe Systems, Mountain View, CA, United States) was used to process the images obtained by light and confocal laser scanning microscopy. Care was taken to keep the same brightness, sharpness, color saturation, and contrast in the various pictures.

### Quantification of Neuronal Numbers

For quantification of neuronal numbers, images of immunohistochemical labelings were taken at a Keyence BZ-9000 microscope. The outline of hippocampal layers, the location of neurons inside these layers and quantitative analysis of the proportion of hAPP-immunoreactive interneuron populations were assessed by means of BZ-II Analyzer software (Keyence).

The expression of NeuN/HuCD, hAPP, parvalbumin, calbindin, calretinin, and somatostatin-positive neurons was investigated in both hippocampi of all animals. Each section was first viewed at low magnification (5×) for outlining the relevant hippocampal layers, and dissector frames were placed in a systematic consecutive fashion in the delineated regions of the sections. On average the post-processing shrinkage of the tissues resulted in a final section thickness of about 16 μm, which permitted a consistent sampling of 10 μm with the dissector and the use of guard zones of 2 μm on either sides of the section. All neurons that fell within the dissector frames were then counted at higher magnification (10×). In all sections analyzed, the total number of neurons in each hippocampal layer was set to 100%. The proportion of hAPP-immunoreactive neurons and neurons immunoreactive for interneuron markers was calculated and statistical differences were evaluated by unpaired *t* test.

## Results

The hAPP-specific 1D1 antibody used was shown by us to specifically detect the N-terminus of human, but not endogenous mouse APP in mouse brain sections (Höfling et al., 2016). In control experiments of the present study we included wild type mouse brain sections as negative control to validate specific hAPP transgene detection. In preceding experiments, we tested the 1D1 antibody at different concentrations on hAPP-transgenic Tg2576 and wild type mouse brain sections. With the highest 1D1 concentration not generating labeling in wild type mouse brain, we still observed the same pattern of labeling of hippocampal interneurons as reported below. The hippocampal formation can be subdivided into DG and hippocampus proper consisting of segments *cornu ammonis* (CA) 1, 2, 3, and 4. In addition, hippocampal layers with defined neuronal populations can be distinguished along these hippocampal subfields. In order to obtain highly detailed information on the specific distribution of hAPP expressing interneurons, our analyses were performed in hippocampal layers excluding pyramidal and granule cell layers. We observed hAPP expression in subsets of hippocampal interneurons ranging between 57 and 100% in hippocampal layers analyzed (Table 1). In contrast, endogenous mouse APP is ubiquitously expressed by virtually all neurons (Buxbaum et al., 1993; Neve et al., 1996) and critical for neuronal development and viability as well as for synapse formation and stabilization (Priller et al., 2006). Thus, endogenous mouse APP is expressed by almost all hippocampal neurons, whereas transgene expression of hAPP is limited to subsets of hippocampal neurons.

**Table 1:** Proportions of hAPP-positive neurons in hippocampal layers and proportions of hAPP-positive neurons expressing parvalbumin, calbindin, calretinin, and somatostatin.

	hilus	S. mol.	S. lac.	S. rad.	S. ori.
	% mean ± SEM				
hAPP/neurons	59.5 ± 15.0	100 ± 0	100 ± 0	63.6 ± 8.2	56.7 ± 3.4
parvalbumin/hAPP	3.7 ± 0.3	0.6 ± 0.2	0	4.0 ± 1.0	24.8 ± 3.6
calbindin/hAPP	1.2 ± 0.9	2.4 ± 0.2	4.9 ± 2.1	20.1 ± 9.6	41.3 ± 8.0
calretinin/hAPP	18.4 ± 6.4	3.3 ± 1.5	4.8 ± 1.5	4.3 ± 1.8	8.3 ± 2.1
somatostatin/hAPP	30.2 ± 4.6	0.1 ± 0.1	0.3 ± 0.3	10.7 ± 4.2	34.6 ± 2.5

*S. mol., stratum moleculare; S. lac., stratum lacunosum moleculare; S. rad., stratum radiatum; S. ori., stratum oriens.*

### GABAergic Interneurons in Hippocampal Layers of Wild Type and Tg2576 Mice

**FIGURE 1:**
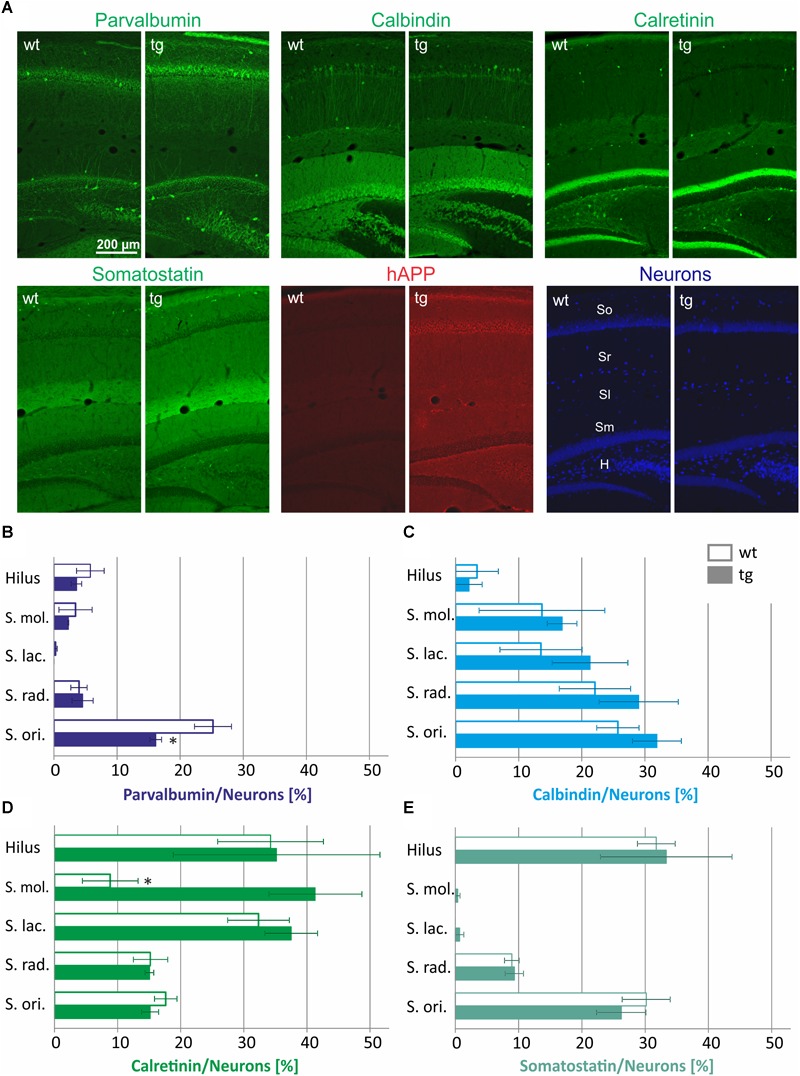
Quantification of interneuron numbers in hippocampal layers of wild type (wt) and hAPP-transgenic Tg2576 mice (tg). In panel **(A)** typical staining patterns of parvalbumin-, calbindin-, calretinin-, somatostatin-, and hAPP-immunoreactive neurons are depicted in wt and tg mouse hippocampus. Note the similar labeling intensity in wt and tg brain sections for all antigens except hAPP. The hippocampal layers analyzed are indicated in the wt pan-neuronal labeling image (Neurons). In panels **B–E**, the proportions of parvalbumin-, calbindin-, calretinin-, and somatostatin-positive neurons in hippocampal layers of wt and tg mice are quantified. Total neuronal numbers in each layer (hilus 69.8 ± 2.8; S. mol 48.2 ± 6.7; S. lac. 68.5 ± 8.5; S. rad. 407.2 ± 49.0; S. ori. 269.5 ± 13.0) were set to 100%. Note the similar interneuron proportions in wt and tg hippocampus for most data analyzed. ^∗^significantly different from wild type (*p* < 0.05; unpaired *t* test).

First, we tested whether the numbers of CBP and somatostatin-expressing interneurons is similar in hippocampal layers of 3-month-old wild type and hAPP-transgenic Tg2576 mice. Since the absolute number of neurons and the neuronal number per area differ strongly along the rostro-caudal extension of the hippocampal formation, the proportion of CBP- and somatostatin-containing neurons of all neurons was calculated in brain sections at a narrow brain cutting level of bregma -2.70 to Bregma -3.16 mm by immunofluorescent co-labeling of the CBP and somatostatin with a combination of the pan-neuronal markers NeuN/HuCD (Figure 1). Typical examples of these labelings of wild type and Tg2576 hippocampus are displayed in Figure 1A. Visual examination of the staining patterns indicated a similar appearance of all CBP analyzed as well as of somatostatin, while hAPP immunoreactivity was absent in wild type brain sections. Data of the quantitative analyses of these labelings are depicted in Figure 1B–E.

As to the expression of the different neuropeptides we detected striking differences in the hippocampus layer-specific abundance of CBP and somatostatin-positive neurons in wild type mice. For example, approximately 25% of neurons in *stratum oriens* were *parvalbumin*-immunoreactive, whereas only 5% in *stratum radiatum, stratum moleculare* and the hilus of DG displayed parvalbumin immunoreactivity and *stratum lacunosum moleculare* was completely devoid of parvalbumin neurons (Figure 1B). *Calbindin*-expressing neurons were highly abundant in *stratum oriens* and *stratum radiatum* (30% of all neurons) and to a lesser extent in neurons of *stratum moleculare* and *stratum lacunosum moleculare* (15% of all neurons), whereas in hilus of DG only 5% of all neurons expressed this protein (Figure 1C). An inverse pattern in the abundance across hippocampal layers was detected for *calretinin*-containing interneurons. Here, more than 30% of all neurons in the hilus of DG and in *stratum lacunosum moleculare* were calretinin-immunoreactive, whereas only 15% of neurons in *stratum radiatum* and *stratum oriens* and less than 10% of neurons in *stratum moleculare* were calretinin-immunoreactive (Figure 1D). Somatostatin-containing neurons were most abundant in *stratum oriens* and hilus of DG (30% of all neurons), followed by *stratum radiatum* (10% of all neurons) and were found to be absent in DG molecular layer and in *stratum lacunosum moleculare* (Figure 1E).

For most labelings, there were no statistically significant differences between wild type and Tg2576 mice in the abundance of parvalbumin-, calbindin-, calretinin-, or somatostatin-positive neurons in hippocampal layers analyzed (Figure 1B–E). The only exceptions are a lower proportion of parvalbumin-containing neurons in *stratum oriens* (Figure 1B) and a higher proportion of calretinin-immunoreactive neurons in *stratum moleculare* of Tg2576 mice (Figure 1D). Thus, in general, the appearance and distribution of GABAergic interneurons in hippocampus of young Tg2576 mice is preserved compared to the wild type.

### Human APP Transgene Expression by Hippocampal GABAergic Interneurons

The transgenic hAPP was found to be expressed by interneurons across all hippocampal layers. We, therefore, wanted to analyze the hAPP transgene expression by hippocampal GABAergic interneurons in detail. In particular, the following questions were addressed. (i) Which proportion of hAPP-expressing hippocampal interneurons is immunoreactive for parvalbumin, calbindin, calretinin, and somatostatin in defined hippocampal layers? (ii) Which proportion of parvalbumin, calbindin, calretinin, and somatostatin-positive neurons expresses hAPP?

**FIGURE 2:**
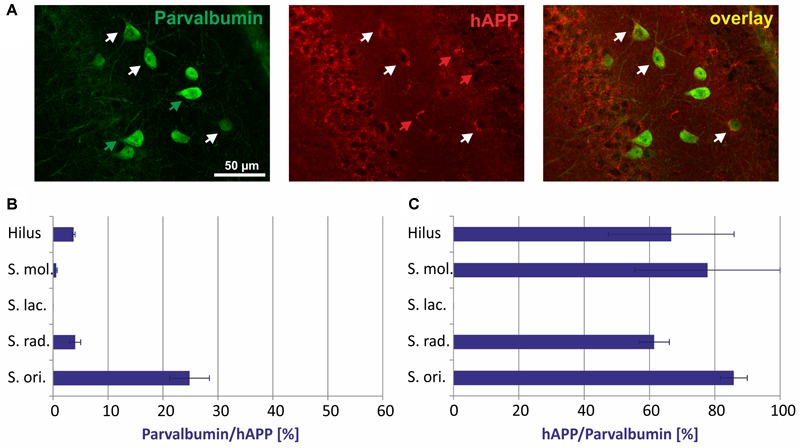
Quantitative analyses of the co-localization between parvalbumin-immunoreactive neurons and hAPP-expressing interneurons in hippocampus of Tg2576 mice. Typical examples of double labelings in *stratum oriens* are shown in panel **(A)**. White arrows point to double-labeled neurons, green, and red arrows to neurons that are only labeled by green (parvalbumin) and red (hAPP) fluorescence, respectively. Quantitative analysis revealed that of all hAPP transgene-expressing interneurons 0% (in *stratum lacunosum moleculare*) to 25% (in *stratum oriens*) were parvalbumin-immunoreactive **(B)**. Of all parvalbumin-expressing neurons, 60 to 80% expressed the hAPP transgene in hilus of DG, *stratum moleculare, stratum radiatum*, and *stratum oriens* **(C)**.

**FIGURE 3:**
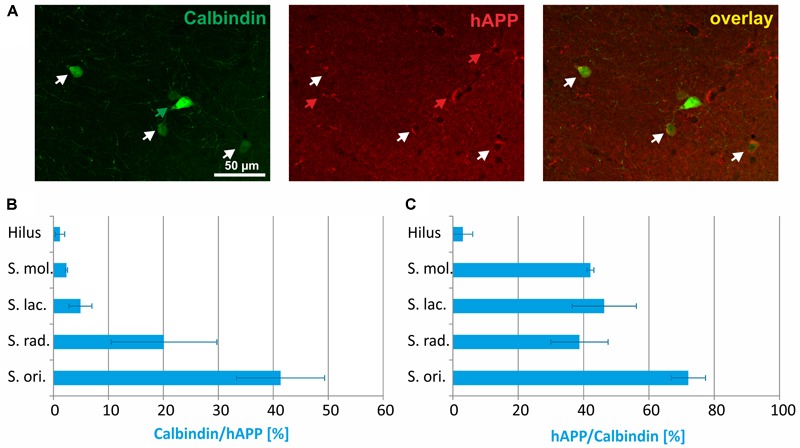
Quantitative analyses of the co-localization between calbindin-immunoreactive neurons and hAPP-expressing interneurons in hippocampus of Tg2576 mice. Typical examples of double labelings in *stratum radiatum* are shown in panel **(A)**. White arrows point to double-labeled neurons, green, and red arrows to neurons that are only labeled by green (calbindin) and red (hAPP) fluorescence, respectively. Quantitative analysis revealed that of all hAPP transgene-expressing interneurons 2% (in DG hilus) to 42% (in *stratum oriens*) were calbindin-immunoreactive **(B)**. Of all calbindin-expressing neurons, 5 to 70% expressed the hAPP transgene **(C)**.

**FIGURE 4:**
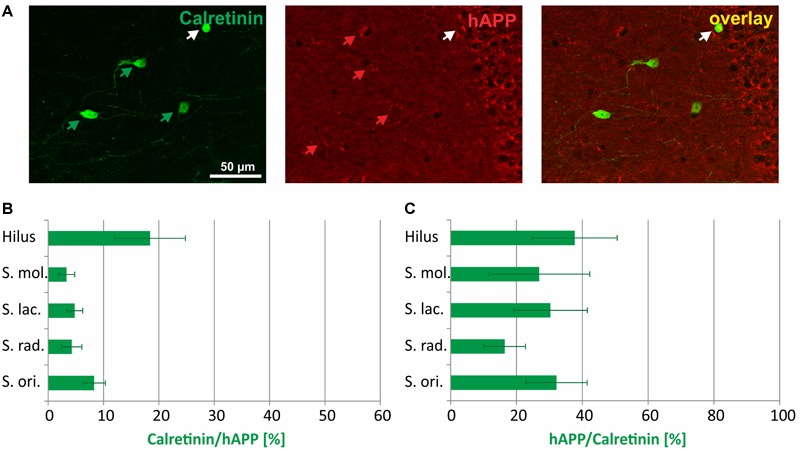
Quantitative analyses of the co-localization between calretinin-immunoreactive neurons and hAPP-expressing interneurons in hippocampus of Tg2576 mice. Typical examples of double labelings in *stratum radiatum* are shown in panel **(A)**. White arrows point to double-labeled neurons, green, and red arrows to neurons that are only labeled by green (calretinin) and red (hAPP) fluorescence, respectively. Quantitative analysis revealed that of all hAPP transgene-expressing interneurons 4% (in *stratum moleculare*) to 18% (in DG hilus) were calretinin-immunoreactive **(B)**. Of all calretinin-expressing neurons, 18 to 38% expressed the hAPP transgene in defined hippocampal layers **(C)**.

**FIGURE 5:**
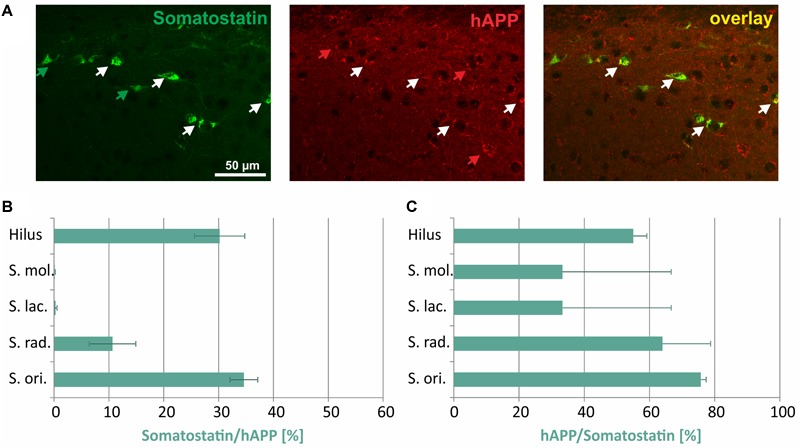
Quantitative analyses of the co-localization between somatostatin-immunoreactive neurons and hAPP-expressing interneurons in hippocampus of Tg2576 mice. Typical examples of double labelings in *stratum oriens* are shown in panel **(A)**. White arrows point to double-labeled neurons, green, and red arrows to neurons that are only labeled by green (somatostatin) and red (hAPP) fluorescence, respectively. Quantitative analysis revealed that of all hAPP transgene-expressing interneurons 1 to 2% in *stratum moleculare* and *stratum lacunosum moleculare* to approximately 30% (in DG hilus and *stratum oriens*) were somatostatin- immunoreactive **(B)**. Of all somatostatin-expressing neurons, 30 to 76% expressed the hAPP transgene in all hippocampal layers **(C)**.

In general, there was no GABAergic interneuron subpopulation that did not express the hAPP transgene. On the other hand, in no case all neurons of such a subpopulation expressed hAPP (Figure 2–5). Typical examples of parvalbumin/hAPP double labelings in *stratum oriens* are shown in Figure 2A. Quantitative analysis revealed that of all hAPP transgene-expressing interneurons in *stratum oriens*, 25% were *parvalbumin*-immunoreactive, whereas in *stratum radiatum* and in hilus of DG only 5% and in *stratum moleculare* only 2% of hAPP-positive neurons displayed parvalbumin immunoreactivity. *Stratum lacunosum moleculare* did not have any neurons expressing both hAPP and parvalbumin (Figure 2B). On the other hand, of all parvalbumin-expressing neurons, 60 to 80% expressed the hAPP transgene in hilus of DG, *stratum moleculare, stratum radiatum*, and *stratum oriens*, respectively (Figure 2C).

Double immunofluorescent labelings for calbindin and hAPP in *stratum radiatum* are shown in Figure 3A. The proportion of hAPP expressing neurons positive for *calbindin* differed considerably between layers and in hippocampus proper amounted to 42% in *stratum oriens*, 20% in *stratum radiatum*, and 5% in *stratum lacunosum moleculare*, respectively, and in DG to 3% in *stratum moleculare* and 2% in the hilus (Figure 3B). Of all calbindin-expressing neurons, 70% expressed hAPP in *stratum oriens* and approximately 40% in *stratum moleculare, stratum lacunosum moleculare*, and *stratum radiatum*, whereas less than 5% of calbindin-positive neurons in the hilus of DG expressed hAPP (Figure 3C).

The immunohistochemical co-localization of calretinin and hAPP in *stratum radiatum* is shown in Figure 4A. The highest proportion of hAPP expressing neurons that was positive for *calretinin* was detected in the hilus of DG (18%), followed by *stratum oriens* (8%), *stratum lacunosum moleculare* (6%), *stratum radiatum* (5%), and *stratum moleculare* (4%) (Figure 4B). Across hippocampal layers, the proportion of calretinin-positive neurons expressing the hAPP transgene was rather homogenous, reaching 30 to 40% in hilus, and *stratum moleculare* of DG as well as *stratum lacunosum moleculare* and *stratum oriens* and 16% in *stratum radiatum* of hippocampus proper (Figure 4C).

In Figure 5A, typical examples of somatostatin/hAPP double immunofluorescent labelings in *stratum oriens* are displayed. Of all hAPP-positive neurons 30% in hilus of DG and 35% in *stratum oriens* were immunoreactive for *somatostatin*, whereas only 10% of hAPP-expressing neurons in *stratum radiatum* and 2% in *stratum moleculare* and in *stratum lacunosum moleculare* displayed somatostatin immunoreactivity (Figure 5B). In all hippocampal layers the proportion of somatostatin expressing neurons which was positive for the hAPP transgene was at least 25% (*stratum moleculare, stratum lacunosum moleculare*) and reached around 60% in hilus of DG and *stratum radiatum*, respectively, and 76% in *stratum oriens* (Figure 5C).

For a general overview, the proportions of hAPP-positive neurons expressing parvalbumin, calbindin, calretinin, and somatostatin across hippocampal layers are displayed in Table 1 and the proportions of total parvalbumin-, calbindin-, calretinin-, and somatostatin-positive neurons expressing hAPP are given in Table 2.

**Table 2:** Proportions of parvalbumin-, calbindin-, calretinin-, and somatostatin-positive neurons expressing hAPP.

	Hilus	S. mol.	S. lac.	S. rad.	S. ori.
	% mean ± SEM				
hAPP/parvalbumin	66.7 ± 19.2	77.8 ± 22.2	0	61.5 ± 4.7	85.8 ± 4.1
hAPP/calbindin	3.0 ± 3.0	42.1 ± 1.1	46.3 ± 9.8	38.7 ± 8.8	72.1 ± 5.3
hAPP/calretinin	37.7 ± 12.9	26.9 ± 15.4	30.3 ± 11.2	16.4 ± 6.3	32.2 ± 9.3
hAPP/somatostatin	55.1 ± 4.2	33.3 ± 33.3	33.3 ± 33.3	64.0 ± 14.7	75.7 ± 1.6

*S. mol., stratum moleculare; S. lac., stratum lacunosum moleculare; S. rad., stratum radiatum; S. ori., stratum oriens.*

**FIGURE 6:**
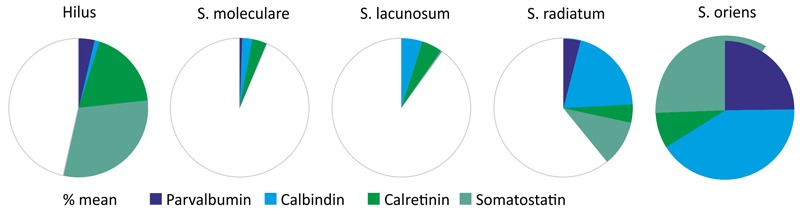
In this Figure, the proportion of hAPP-positive hippocampal interneurons co-expressing parvalbumin, calbindin, calretinin, and somatostatin is summed up in individual hippocampal layers. In *stratum moleculare* and *stratum lacunosum moleculare* less than 10% of all hAPP-expressing neurons are immunoreactive for the four interneuron markers analyzed, whereas in *stratum radiatum* and DG hilus 40 to 50%, and in *stratum oriens* all hAPP-immunoreactive neurons are positive for these interneuron markers. Additionally, the contribution of individual interneuron types to the hAPP-expressing population varies considerably in hippocampal layers as indicated by the respective color.

When the proportion of hAPP-positive hippocampal interneurons co-expressing parvalbumin, calbindin, calretinin, and somatostatin is summed up in individual hippocampal layers, clear differences in hAPP transgene expression by these GABAergic interneurons can be established. In *stratum oriens*, the highest proportion of hAPP-expressing neurons was found to be immunoreactive for the interneuron markers tested (Figure 6). Among the hAPP-positive neurons in *stratum oriens*, most were immunoreactive for calbindin and somatostatin, respectively, followed by parvalbumin and calretinin (Figure 6). In contrast, in *stratum moleculare* and in *stratum lacunosum* moleculare less than 10% of hAPP-positive neurons expressed one of the markers investigated. Among those, most were immunoreactive for calbindin and calretinin but almost none for parvalbumin and somatostatin (Figure 6). In *stratum radiatum*, all four interneuron populations contributed to a total 40% of hAPP-expressing neurons, with calbindin and somatostatin-positive neurons having the biggest impact.

Thus, in defined hippocampal layers there are robust differences in CBP and somatostatin expression by hAPP-positive neurons ranging from 10% in *stratum moleculare* and *stratum lacunosum moleculare* to 100% in *stratum oriens*. Additionally, the proportion of specific GABAergic interneuron subtypes varies considerably in hippocampal layers with a large population of parvalbumin-positive hAPP neurons in *stratum oriens*, calbindin-positive hAPP neurons in *stratum oriens* and *stratum radiatum*, calretinin-positive hAPP neurons in hilus of DG and somatostatin-positive hAPP neurons in *stratum oriens* as well as in hilus of DG. It remains an open question why the promoter driving transgene expression is only active in a subset of the hippocampal interneuron populations investigated here. *Vice versa*, there is a high proportion of hAPP-positive neurons that does not express any of the interneuron markers studied; in particular in *stratum moleculare* and *stratum lacunosum moleculare*. These neurons apparently are subpopulations of hippocampal interneurons other than the ones depicted by CBP and somatostatin immunoreactivity. They might be distinguished based on different characteristics like morphology, wiring and neurochemical properties such as expression of neuropeptides, enzymes and receptors.

## Discussion

Many experimental studies using APP-transgenic AD animal models suffer from a lack of information on the distinct brain region and cell type-specific transgene expression patterns. These data are, however, the pre-requisite to understand pathogenic mechanisms on the subcellular, cellular, neuronal network and systemic level and to allow comparison of experimental outcome obtained from different transgenic mouse lines. Our data presented here indicate that defined interneuron populations in hippocampus expressing transgenic hAPP to different extents may selectively contribute to aspects of pathological disturbances and learning and memory deficits well before amyloid plaque pathology in Tg2576 mice. We decided to analyze young Tg2576 mice before the onset of amyloid pathology for hAPP expression because in older mice the affected neurons may have degenerated and thus escaped quantification.

### Parvalbumin

In CA1 of hippocampal slices of 6- to 7-month-old Tg2576 mice LTP was found to be impaired, but restored in Tg2576 mice aged 14 to 19 months (Huh et al., 2016). The authors explained these findings with the selective loss of inhibitory parvalbumin-positive neurons in hippocampus during aging, which resulted in enhanced excitatory signaling (Huh et al., 2016). This notion is supported by findings on the critical role of parvalbumin interneurons for the down regulation of LTP at Schaffer collateral-CA1 synapses (Chen et al., 2010). Using resting-state functional MRI to assess brain functional connectivity in Tg2576 mice, network hypersynchrony was detected in hippocampus of 3- to 5-month-old Tg2576 mice but an age-dependent reduction in hippocampal functional connectivity was revealed in 8- and 18-month-old Tg2576 mice (Shah et al., 2016). Interestingly, in another hAPP-transgenic mouse model spontaneous epileptiform discharges induced by network hypersynchrony were attributed to impaired function of parvalbumin neurons (Verret et al., 2012). Our data suggest that hAPP transgene expression by parvalbumin-positive hippocampal GABAergic interneurons may contribute to the described impairments of neuronal network activity, LTP and, subsequently, hippocampus-dependent spatial learning. Moreover, Abeta deposits in old Tg2576 mice appear primarily in *strata oriens, lacunosum moleculare* and *moleculare* (Hartlage-Rübsamen et al., 2011), indicating a contribution of transgene expression by these neuronal populations to Abeta pathology. Similar conclusions have been drawn in a study using APP/PS1 mice, where reductions in parvalbumin, calbindin, and calretinin immunoreactivity in DG were more pronounced at Bregma levels with high Abeta plaque load than at Bregma levels with low plaque load (Popović et al., 2008).

### Calbindin

The population of calbindin-immunoreactive neurons was demonstrated to be stable during aging in hippocampus of wild type mice between 3 and 18 months (Krezymon et al., 2013). In male Tg2576 mice, however, there was an age-dependent decline of calbindin immunoreactivity in the DG molecular layer resulting in significantly reduced calbindin immunoreactivity of 12- and 18-month-old Tg2576 mice compared to wild type mice. Preceding these changes in DG calbindin immunoreactivity, severe disturbances in hippocampal neurogenesis of Tg2576 mice were observed which compromised functional integration of new neurons into hippocampal circuits (Krezymon et al., 2013). Implications for a disturbed hippocampal neurogenesis were also obtained in the APPswe/PS1dE9 transgenic mouse model by reduced numbers of calretinin-positive and doublecortin-positive neurons in the subgranular zone of the DG hilus compared to wild type mice (Verdaguer et al., 2015). Moreover, in hAPP_FAD_ mice, learning deficits correlated strongly with decreased levels of calbindin in the DG (Palop et al., 2003) and epigenetic suppression of hippocampal calbindin results in disturbance of spatial memory, whereas restoring calbindin expression in these mice improves performance in spatial memory (You et al., 2017).

### Calretinin

In contrast to the high vulnerability of hippocampal calbindin-positive neurons in the human AD brain and its animal models, calretinin-positive fibers in proximity of Abeta plaques were shown to be less affected by dystrophic morphology than neurites containing neurofilament-triplet proteins in Tg2576 and APPswe/PS1dE9 mice and human AD affected brains (Mitew et al., 2013). However, the number of calretinin-positive interneurons is profoundly decreased in CA1 and CA2/3 hippocampal subfields of APP/PS1 mice at a very early stage of pathology, whereas hippocampal calretinin neurons belonging to the Caja-Retzius cells were not affected (Baglietto-Vargas et al., 2010). Interestingly, the early and selective calretinin cell loss preferentially occurred in parallel to the appearance of Abeta deposits in axonal terminal fields of these neurons (Baglietto-Vargas et al., 2010). Thus, the calretinin-positive population of hippocampal interneurons appears to be differentially affected by Abeta pathology.

### Somatostatin

In addition to CBP-positive interneurons, a subpopulation of GABAergic hippocampal interneurons expressing the peptide hormone somatostatin has been demonstrated to be critically involved in learning and memory processes (Lovett-Barron et al., 2014), most likely by providing feedback inhibition to CA1 pyramidal neurons (Royer et al., 2012). In hippocampus of APP/PS1-transgenic mice, an early and substantial reduction of somatostatin-positive neurons, which correlated with Abeta pathology, was observed in hippocampal subfields CA1, CA2/3, and in DG (Ramos et al., 2006). The dysfunction of these neurons in APP/PS1 mice was shown to be associated with memory deficits (Schmid et al., 2016). Also in Tg2576 mice a significant reduction in the number of somatostatin-positive interneurons in *stratum oriens* of CA1 was reported and related to impairments of performance in contextual fear and spatial memory tasks (Perez-Cruz et al., 2011). Another line of evidence for the importance of somatostatin-positive hippocampal interneuron function for learning processes arises from pharmacological studies using somatostatin receptor 4 agonists (Sandoval et al., 2013). When Tg2576 mice were treated with the selective somatostatin receptor 4 agonist NNC 26-9100, learning improvements and reductions in oligomeric Abeta concentrations were observed (Sandoval et al., 2013).

### Relation to AD

The presented data are relevant to the understanding of different aspects of synaptic, neuronal and network dysfunction in hippocampus of young and aged Tg2576 mice as well as systemic deficits in learning and memory and the contribution of defined hippocampal GABAergic subpopulations to these pathogenic processes. However, no direct conclusions can be drawn with regard to the involvement of these neuronal populations in AD pathogenesis because a higher, or lower, proportion of these neurons may express varying amounts of APP and produce pathogenic Abeta assemblies. Nevertheless, in the APP/PS1 model, the reduction of the number of parvalbumin expressing neurons in the CA1/2 hippocampal sublayer and of calretinin-immunoreactive neurons in DG is at the same order of magnitude as in *post mortem* hippocampal tissue from AD subjects (Takahashi et al., 2010). Reductions in the number of hippocampal parvalbumin-immunoreactive neurons in AD were already reported by Brady and Mufson (1997). They observed an up to 60% decrease in the number of parvalbumin-positive neurons in CA1/2/4 and DG, but not in CA3, subiculum, or presubiculum. They concluded, that the selective vulnerability of subsets of hippocampal parvalbumin neurons in AD may be related to differential connectivity. Accordingly, DG and CA1 parvalbumin-positive neurons connected to the cerebral cortex would be more vulnerable than neurons located in the subiculum or presubiculum, respectively, which are connectionally related to subcortical areas (Brady and Mufson, 1997). Hippocampal calbindin-immunoreactive neurons, on the other hand, were reported to be spared from neurofibrillary tangle formation and appeared to be unaffected by Abeta pathology at early stages of AD, whereas this resistance is lost at advanced disease stages (Iritani et al., 2001). Calbindin immunoreactive neurons in DG were also reported to be differentially affected in AD and other neurodegenerative disorders (Stefanits et al., 2014). The proportion of calbindin-negative DG granule neurons was found to increase with Braak stage of AD and was significantly higher than in Creutzfeldt-Jakob disease, argyrophilic grain disease, frontotemporal lobar degeneration-TDP types A and B, but not compared to frontotemporal lobar degeneration-Tau Pick’s disease type (Stefanits et al., 2014). Also in entorhinal cortex, a major source of DG innervation, a differential impact of AD pathology on neurons expressing CBPs was reported (Mikkonen et al., 1999). Non-principal cells containing parvalbumin or calbindin were affected at early disease stages, whereas non-principal neurons containing calretinin were more viable, even in AD cases with severe entorhinal pathology (Mikkonen et al., 1999).

### Non-neuronal hAPP Expression

In a recent study we demonstrated that hAPP expression is not neuron-specific but also detectable in subsets of resting astrocytes in neocortex and corpus callosum of Tg2576 mice before the onset of amyloid plaque formation (Heiland et al., 2019). In hippocampus, the majority of hAPP immunoreactivity arises from neurons, but there is also hAPP immunoreactivity present in glial cells of astrocytic morphology [see filmstrip links provided in Heiland et al. (2019)]. Although the proportion of astrocytic hAPP expression is low, we cannot rule out an astrocytic contribution to impairments in spatial navigation, learning, memory, hippocampal LTP formation, and neuronal network activity induced by Abeta or C-terminal APP fragments. Also in human brain, non-neuronal expression of APP mRNA has been demonstrated and related to pathology (Golde et al., 1990).

## Conclusion

In this study, hAPP transgene expression by different populations of GABAergic interneurons in defined hippocampal layers of Tg2576 mice was investigated for the first time. Our results partly explain disturbed hippocampus-dependent processes and functions such as LTP, neuronal network activity, learning and spatial memory and neurogenesis. Thus, the detailed analysis of transgene expression patterns allows revealing pathological mechanisms and obtaining novel insights in hAPP-dependent disturbances in brain. Technical aspects of transgenic animal generation such as transgene isoform and promoter selection, the genomic integration of the transgene, co-pathologies and compensatory mechanisms result in strikingly different transgene expression patterns in different mouse lines (Höfling et al., 2016). We, therefore, strongly recommend carefully analyzing and comparing transgene expression patterns in different mouse lines used as animal models to mimic amyloid pathology of AD.

## Author Contributions

CH designed the study, carried out the experiments, analyzed the data, and drafted the manuscript. ES carried out the experiments and analyzed the data. P-HK and SL participated in designing the experiments and drafted the manuscript. MH-R designed the study, analyzed the data, and drafted the manuscript. SR conceived, designed and coordinated the study and drafted the manuscript. All authors gave final approval for publication.

## Conflict of Interest Statement

The authors declare that the research was conducted in the absence of any commercial or financial relationships that could be construed as a potential conflict of interest.
